# The training and expectations of medical students in Mozambique

**DOI:** 10.1186/1478-4491-5-11

**Published:** 2007-04-19

**Authors:** Fernando Sousa, João Schwalbach, Yussuf Adam, Luzia Gonçalves, Paulo Ferrinho

**Affiliations:** 1 Associação para o Desenvolvimento e Cooperação Garcia de Orta (AGO), Lisbon, Portugal; 2 Unidade de Epidemiologia e Bioestatistica, Instituto de Higiene e Medicina Tropical, Universidade Nova de Lisboa, Lisbon, Portugal; 3 Unidade de Sistemas de Saúde e Centro de Malária e Outras Doenças Tropicais, Instituto de Higiene e Medicina Tropical, Universidade Nova de Lisboa, Lisbon, Portugal

## Abstract

**Background:**

This paper describes the socio-economic profile of medical students in the 1998/99 academic year at the Universidade Eduardo Mondlane (UEM) Medical Faculty in Maputo. It aims to identify their social and geographical origins in addition to their expectations and difficulties regarding their education and professional future.

**Methods:**

The data were collected through a questionnaire administered to all medical students at the faculty.

**Results:**

Although most medical students were from outside Maputo City and Maputo Province, expectations of getting into medical school were already associated with a migration from the periphery to the capital city, even before entering medical education. This lays the basis for the concentration of physicians in the capital city once their term of compulsory rural employment as junior doctors is completed.

The decision to become a doctor was taken at an early age. Close relatives, or family friends seem to have been an especially important variable in encouraging, reinforcing and promoting the desire to be a doctor.

The academic performance of medical students was dismal. This seems to be related to several difficulties such as lack of library facilities, inadequate financial support, as well as poor high school preparation.

Only one fifth of the students reported receiving financial support from the Mozambican government to subsidize their medical studies.

**Conclusion:**

Medical students seem to know that they will be needed in the public sector, and that this represents an opportunity to contribute to the public's welfare. Nevertheless, their expectations are, already as medical students, to combine their public sector practice with private medical work in order to improve their earnings.

## Background

Mozambique, previously a Portuguese colony, became independent in 1975 and had a single party political system until 1994, when the first multi-party elections were held.

Mozambique is classified as a low human development country and the poverty index is the highest in the Southern African Development Community (SADC) region [1,2].

Since the peace agreement signed by Resistência Nacional Moçambicana (RENAMO) and Frente de Libertação de Moçambique (FRELIMO) in 1992, Mozambique has embarked on a major economic restructuring process, changing from a centrally planned to a market economy [3]. A new constitution was introduced in 1990, opening the way for the peace process and for a multi-party election in 1994. A plethora of new laws and regulations have been issued since then, legalizing or liberalizing economic activities including health services that previously were under absolute state control [2].

Following the civil war, the health services have gone through a period of rapid expansion but the access to health care is still poor [4]. In 1999, of a total of 406 Medical doctors holding clinical posts, there were 204 foreigners. Of 298 specialist medical doctors, 173 were concentrated in Maputo city (responsible for over 34% of the national Gross Domestic Product [1]) where it is easier to develop private medical practice. According to Vio, many of the national doctors work part-time in the private sector [5].

Currently, the Mozambican health system is a mixed economy of public and private sector players. The public healthcare sector actually involves eight Ministries, but it is dominated by the services provided by the Ministry of Health [4], the main provider of health care services in the country which remains highly dependent upon external financial support [5].

In Mozambique medical students are trained in two faculties, the Maputo based, public sector Medical Faculty of the University Eduardo Mondlane and the private sector Faculty of Medicine in Beira, integrated into the Catholic University. There is talk of a third Faculty in Nampula. The Beira Faculty of Medicine is a recently established institution, functioning since 2001.

**Figure 1:**
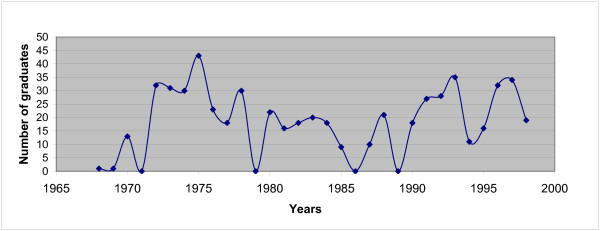
**Number of graduates of the Maputo Medical School- UEM**. Source: Medical Faculty of Maputo

The principal provider of undergraduate medical training has been the Faculty of Medicine in Maputo. Its output has been erratic (see Figure 1). Medical education has tried to keep up with the changes in the health care system. Established in 1963 in the colonial period, it has, since independence, trained doctors to meet to some extent the needs of a then exclusively public sector socialist health care system, partially free at the point of delivery. More recently, the Medical School has tried to adapt its medical syllabus to accommodate a more nuanced and realistic vision of a Mozambican society with a multitude and diversity of health care sectors [2].

The training curriculum introduced after independence remained unchanged up to 1982. In 1985, the teaching of several ideological subjects (Marxism-Leninism, and Political Economy) was dropped. The course duration was increased from six to seven years. New subjects were introduced such as Informatics, English and Physical Education. These three subjects were subsequently dropped during a period of curriculum reform in 1995/96. A new curriculum planned in the "2003–2005 Strategic Plan of the Faculty" is currently being implemented [2].

Concerning the selection of medical students, there are – in several countries including some in Africa – programmes based on affirmative action aiming to increase the intake of medical students from disadvantaged socioeconomic, ethnic, or geographic factions [6]. The purpose of these programmes is to redress inequities from the past, avoiding in particular geographical imbalances [7], especially in rural or poor areas. Such imbalances result in a situation that has serious adverse consequences for health system performance [8]. On the other hand, the programmes are designed to select applicants who have genuine merit, in order to produce physicians that reflect more "closely the social groups for which they are going to care" [7]. Nevertheless, most health training institutions, including the Faculty of Medicine in Maputo, still use academic record as the primary selector criteria for medical school entrance.

This paper describes the socio-economic profile of medical students in the 1998/99 academic year at the Universidade Eduardo Mondlane (UEM) Medical Faculty in Maputo, with the aim of identifying their social and geographical origins and their expectations and difficulties regarding their education and professional future.

## Methods

**Figure 2:**
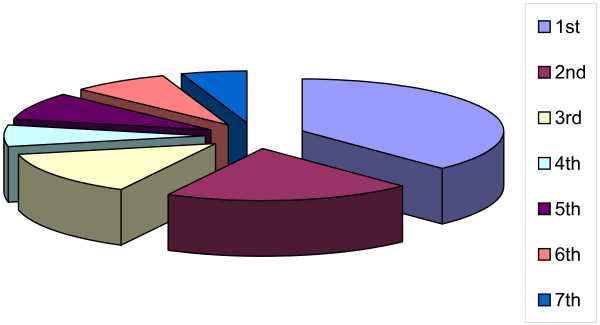
Distribution of all medical students by academic year, 1998/99.

A piloted, standardized questionnaire, with both definite and open-ended questions, was distributed to all registered medical students (from 1 st to 7 th year of medical education) on a specified day, during agreed lecture periods, in April and May of 1999 (see Figure 2).

All data were entered into an Access database and analysed using SPSS. The statistical analysis is mostly descriptive.

**Figure 3:**
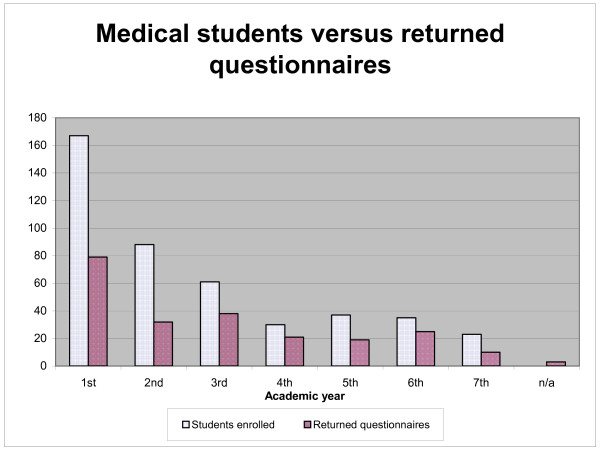
Returned questionnaires from the medical students.

Two hundred and twenty-seven (51%) of the 441 students registered completed and returned the questionnaire (see Figure 3). Their ages ranged from 18 to 36 years (median and mean of 23 years). Sixty-one percent of the respondents were women and 10% were married (86% of those being women).

## Results

This section reports on the students' backgrounds, on the decision to study medicine, on their academic performance and on difficulties and expectations.

### Students' backgrounds

Most (56%) students were born and received their primary school education outside Maputo Province and Maputo City, where the medical school is located. Sixty-three percent of the students enrolled in the medical school had finished their high school education in Maputo, although the region only contains 6% of the country's population. Forty-three percent lived with their parents; 24% with other relatives; 23% in hostels and the remainder indicated other living arrangements.

### The decision to study medicine

Twenty percent took their decision to study medicine when they were aged between 15 to 16 years, although the range reported varied from ages 4 to 30 years. By the age of 18 years, 65% had already decided to undertake a medical course.

Table 1 shows that 90% reported that their parents had in some way been associated with the health sector: as doctors (29%), nurses (29%), health sector personnel (18%), pharmacists (8%), auxiliaries (2%) or in some other category (5%). Forty-six percent reported having uncles and/or aunts that were associated with the health profession, with 24% having friends working in the discipline and 30% noting other reference people similarly involved.

**Table 1:** Students' family, friends, and others associated with the health sector

	**Parents**	**%**	**Friends**	**%**	**Uncles/Aunts**	**%**	**Other**	**%**
**Pharmacists**	18	*8*	6	*3*	13	*6*	4	*2*
**Doctors**	65	*29*	21	*9*	32	*14*	19	*8*
**Nurses**	65	*29*	13	*6*	30	*13*	21	*9*
**Health sector personnel**	40	*18*	11	*5*	20	*9*	12	*5*
**Auxiliaries**	5	*2*	2	*1*	3	*1*	4	*2*
**Other categories**	12	*5*	2	*1*	6	*3*	7	*3*
**No answer**	22	*10*	172	*76*	123	*54*	160	*70*

***Total***	**227**	***100***	**227**	***100***	**227**	***100***	**227**	***100***

The main reasons for choosing medicine as a profession were "to contribute towards the welfare of the public" (60%), "self-realization" (48%), "vocation" (34%) and "social recognition" (13%). "Family tradition" was actually acknowledged as a reason only by 2% of the students.

### Academic performance

Five (6%) of the 79 first-year students were repeating the year for the second or third time. Only 46 (32%) of the 143 students enrolled in the subsequent years had not failed any academic year (see Table 2).

**Table 2:** Academic performance

	**Year of medical degree**							
**Repeating current year of registration for n**^th^**time**	**1**^st^	**2**^nd^	**3**^rd^	**4**^th^	**5**^th^	**6**^th^	**7**^th^	**Total**

**0**	74	19	9	6	3	7	2	*120*
**1**	4	8	13	6	5	9	6	*51*
**2**	1	2	7	6	4	4	1	*25*
**3**		1	5	2	5	3		*16*
**4**		1	4		1	2		*8*
**5**		1						*1*
**6**				1				*1*

***Total***	***79***	***32***	***38***	***21***	***18***	***25***	***9***	***222***

### Financial support

Sixty-nine percent of the students were self-financing their medical education; 19% received a scholarship from the government, 6% from an international NGO and the remainder financed their studies by other means.

### Main difficulties reported

The most frequent difficulties reported by the students during the medical training were: "lack of available reference books" (66%) and "financial" (58%). Other difficulties were "lack of adequate technology" (22%), "teachers not adequately prepared" (22%), "inadequate syllabus" (8%) and "inadequate preparedness by the high school education system" (8%).

### Satisfaction with the academic education received

Fifty-four percent of the students were satisfied or partially satisfied with the burden of lecturing and learning hours demanded by the medical school. Twenty-six percent were unhappy or partially unhappy with it and 20% did not have any opinion.

Regarding the quality of the training received, 52% felt it was adequate or very adequate, 20% that it was inadequate or very inadequate and the remainder did not have any opinion.

### Expectations regarding their future professional income

When asked about their intentions regarding the sectors within which they would like to practice medicine after completing their medical education (more than one choice possible), 82% reported the public sector, 40% the private for profit sector and 21% the private not for profit sector.

Of 186 students who preferred the public sector, 36% indicated the intention of combining a public sector job with work in the private for-profit sector, and 17% declared the intention of coupling public sector activities with activities in the private not-for-profit sector.

**Figure 4:**
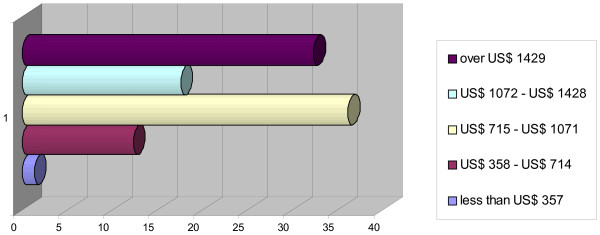
Expectations of future monthly income.

Concerning what they would consider a fair level of monthly income after graduation, the results were: less than US$ 714 for 14%, US$ 715 -1071 for 36%, US$ 1072–1428 for 17%, and 1429 US$ or over for 33% (see Figure 4).

## Discussion

As expected, the medical students questioned were not representative of the diversity of the Mozambican population [9]. Although most were from outside Maputo City and Maputo Province, expectations of being accepted into medical school were already associated with a migration from the periphery to the capital city, even before entering medical education. This forms the basis for the concentration of physicians in the capital city once their term of compulsory rural employment as junior doctors is completed [10].

It is known that an individual's social background, age, gender, individual expectations and career advancement plans influence that person's decisions concerning the geographical location of their medical practice. For example, growing up in rural communities increases the probability of practising in rural areas [6]; female medical doctors are less prone to accept rural posts; and younger individuals with smaller families are more prepared to migrate [8]. The medical faculty's selection criteria do not take such trends into account, although they could help to reduce the concentration of physicians in the capital city.

The decision to become a doctor is taken at an early age. Although this decision seems to be in order to fulfil the students' wishes of contributing to public sector values, it is undeniable that having family and/or friends already in the health professions is likely to have an enormous influence on them. Close relatives or family friends are an especially important variable in encouraging, reinforcing and promoting the desire to be a doctor [9].

The level of academic performance is dismal. This seems to be related to several difficulties such as lack of library facilities, inadequate financial support, as well as poor high school preparation. It is not surprising that poor performance should be associated with a high degree of dissatisfaction with the quality of teaching and burden of lecturing. These difficulties have been previously described [11].

Only one fifth of the students reported receiving financial support from the Mozambican government, a figure that compares unfavourably with the 45% reported for the students who had completed their studies in the previous 5 years [10]. The extent to which this interferes with the ability of students to complete their medical studies or forces them to start the practice of medicine prematurely was not clear.

## Conclusion

Medical students seem to know that they will be needed in the public sector, and that this represents an opportunity to contribute to the public's welfare. Nevertheless, their expectations are, in order to improve their earnings, to combine their public sector practice with private medical work [12,13]. Their income expectations were: for one third of respondents, from US$ 715 to US$ 1071, and for another third, over US$ 1429. These expectations are put into context when one notes that the salary of a newly graduated doctor at the time was about US$ 357a month [14]. Thus, the scene is set for the reality of coping strategies and dual practice that are often unregulated and that plague many countries, including Mozambique [15].

## Competing interests

The author(s) declare that they have no competing interests.
